# The *Drosophila *STIM1 orthologue, dSTIM, has roles in cell fate specification and tissue patterning

**DOI:** 10.1186/1471-213X-8-104

**Published:** 2008-10-24

**Authors:** Jean-Pierre Eid, Alfonso Martinez Arias, Hannah Robertson, Gary R Hime, Marie Dziadek

**Affiliations:** 1 Department of Anatomy and Cell Biology, University of Melbourne, Victoria 3010, Australia; 2 Department of Genetics, University of Cambridge, Cambridge, UK; 3 Australian Research Council Centre of Excellence in Biotechnology and Development, University of Newcastle, Callaghan, NSW 2308, Australia; 4 Cancer Research Program, Garvan Institute of Medical Research, Darlinghurst, NSW, Australia

## Abstract

**Background:**

Mammalian STIM1 and STIM2 and the single *Drosophila *homologue dSTIM have been identified as key regulators of store-operated Ca^2+ ^entry in cells. STIM proteins function both as molecular sensors of Ca^2+^concentration in the endoplasmic reticulum (ER) and the molecular triggers that activate SOC channels in the plasma membrane. Ca^2+ ^is a crucial intracellular messenger utilised in many cellular processes, and regulators of Ca^2+ ^homeostasis in the ER and cytosol are likely to play important roles in developmental processes. STIM protein expression is altered in several tumour types but the role of these proteins in developmental signalling pathways has not been thoroughly examined.

**Results:**

We have investigated the expression and developmental function of dSTIM in *Drosophila *and shown that dSTIM is widely expressed in embryonic and larval tissues. Using the UAS-Gal4 induction system, we have expressed full-length dSTIM protein and a dsRNAi construct in different tissues. We demonstrate an essential role for dSTIM in larval development and survival, and a tissue-specific role in specification of mechanosensory bristles in the notum and specification of wing vein thickness.

**Conclusion:**

Our studies show that dSTIM regulates growth and patterning of imaginal discs and indicate potential interactions with the Notch and Wingless signaling pathways. These interactions may be relevant to studies implicating STIM family proteins in tumorigenesis.

## Background

Ca^2+ ^is a crucial second messenger utilized in a diverse range of cellular processes in the development of multicellular organisms [1,2]. Since changes in cytosolic Ca^2+ ^trigger calcium-dependent cellular responses by activating signaling and transcriptional cascades mediated by Ca^2+^-dependent proteins [3,4], the levels of cytosolic Ca^2+ ^need to be highly regulated to effect appropriate developmental signaling responses. The major intracellular store of Ca^2+ ^is the endoplasmic reticulum (ER) from where Ca^2+ ^is mobilized for signaling processes [5]. Developmental regulation of Ca^2+ ^release from the ER is critical for patterning the body plan of the vertebrate embryo [4]. In dorso-ventral patterning, cytosolic Ca^2+ ^activates protein kinase C and calcium/calmodulin-dependent kinase II enzymes that promote ventral fate through activation of the transcriptional regulator NFAT [6,7], and inhibits dorsal fate by antagonising components of the canonical Wnt/β-catenin pathway [8]. The developmental regulation of the Ca^2+ ^flux between the ER lumen and the cytosol, and the replenishment of Ca^2+ ^stores from extracellular Ca^2+ ^is thus likely to play a significant role in the signaling pathways that specify changes in cell phenotype.

While the IP_3 _receptor-mediated release of Ca^2+ ^from the ER into the cytosol and the SERCA pumps that regulate Ca^2+ ^influx back into the ER are well understood [9], the molecular nature of the components that regulate the replenishment of Ca^2+ ^stores from the extracellular space after depletion of ER stores have only recently been elucidated [10,11]. STIM1 and Orai1 (CRACM1) have been identified as critical regulators of ER Ca^2+ ^homeostasis in mammalian cells [12-16]. STIM1 is predominantly localized in the ER membrane where it functions as the essential molecular sensor of ER Ca^2+ ^levels. When levels are sufficiently depleted STIM1 triggers Ca^2+ ^entry through highly selective store-operated Ca^2+^(SOC) channels in the plasma membrane, also referred to as Ca^2+^-release activated channels (CRAC) in lymphocytes [17]. Ca^2+^-depletion induces rapid aggregation of STIM1 polypeptides within the ER membrane and their subsequent translocation to specific regions of the ER membrane juxtaposed to the plasma membrane [18-22]. At these sites STIM1 activates Ca^2+ ^entry through SOC channels by interacting with the cytosolic domains of Orai1 proteins, the key molecular subunits of SOC channels [23-25]. The structurally related family member STIM2 appears to independently regulate basal ER Ca^2+ ^levels [26] and can also antagonise the function of STIM1 [27]. Both STIM1 and STIM2 have also been implicated in store-independent regulation of Ca^2+ ^entry [28,29], suggesting that STIM proteins may have broad roles in modulating store-operated and store-independent Ca^2+^entry pathways in a variety of cell types.

STIM1 and STIM2 are known to be widely expressed in embryonic and adult tissues [30,31], and recent genetic knockout analyses in mice indicate they have overlapping but not identical functions, and that both are required for survival beyond the first few weeks of life [32]. The important physiological role of STIM-mediated Ca^2+ ^entry through SOC (CRAC) channels has been demonstrated in T lymphocyte activation and contractile function of skeletal muscle [32,33]. STIM1 and STIM2 expression are both critical for SOC influx and NFAT nuclear translocation and cytokine production in mouse T lymphocytes, as well as for the development of regulatory T cells [32]. Defective SOC entry in human lymphocytes results in severe combined immunodeficiency [12]. In murine skeletal muscle STIM1 expression is also required for NFAT-mediated activation of gene expression during myogenesis, with STIM1-deficiency causing skeletal myopathy [33]. These data suggest that STIM proteins might participate in calcium signaling networks by activating calcineurin/NFAT pathways that intersect with other signaling pathways to regulate a wide range of developmental processes [34,35] and are frequently deregulated in tumorigenesis [36]. While NFATc proteins are restricted to vertebrates, calcineurin genes are found in invertebrate organisms such as *Drosophila melanogaster *where they, like their mammalian counterparts, have important roles in muscle development and regulation of the immune response [35,37].

In this study we have utilized *Drosophila *as a model to investigate the role of dSTIM in developmental processes *in vivo*, and to identify potential signaling pathways that intersect with dSTIM function. The single *Drosophila *STIM homologue is structurally similar to both STIM1 and STIM2 [31] and was identified as an essential regulator of SOC entry independently of mammalian STIMs [15]. dSTIM interacts with a single dOrai protein (olf186-F) to activate Ca^2+ ^entry through SOC channels in a similar way to the mammalian counterparts [12,38]. We have generated transgenic *Drosophila *expressing full-length *UAS-dSTIM *cDNA and *UAS-dSTIM *RNAi constructs to determine the effects of STIM1 overexpression and STIM1 knockdown, respectively, in tissues expressing Gal4 under the influence of developmentally regulated cell type-specific promoters [39]. We demonstrate an essential role for dSTIM in larval development and survival, and a tissue-specific role in cell fate specification of mechanosensory bristles in the notum and in specification of wing vein thickness. These studies are the first to demonstrate an important developmental role for the Ca^2+ ^entry regulator STIM1 in tissue patterning.

## Results

### dSTIM expression in early *Drosophila *embryos

**Figure 1:**
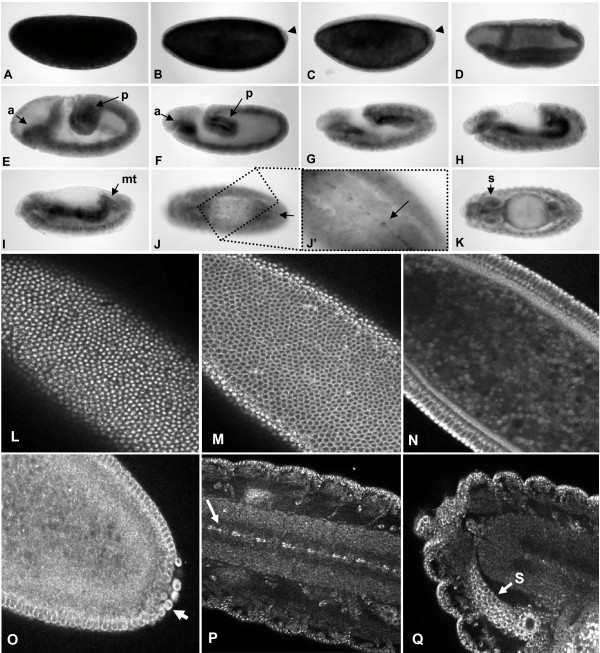
**Embryonic expression of dSTIM**. Whole mount *in situ *RNA hybridization (A-K) and immunofluorescence (L-Q) to detect dSTIM expression at key developmental stages of wild-type *Drosophila *embryogenesis. All embryos are shown with the anterior end to the left, with lateral view shown for all stages 1–12 (A-I, L-O), lateral, ventral and dorsal views for stage 13 (J-K), and ventral view for stage 15 (P, Q). Embryos are staged according to Hartenstein (1993). A) Stage 1 post-fertilisation. B) Stage 4 and C) Stage 5 of cellularisation, arrow indicating pole cells. D) Stage 7 invaginating germ layer. E) Stage 8, and F) Stage 9, showing anterior (a) and posterior (p) midgut primordia. G) Stage 11, beginning of germ-band retraction. H) Stage 12, late midgut fusion. I) Stage 13, lateral view showing malphigian tubules (mt). J) Stage 13, ventral view, showing clusters of dSTIM positive cells along the ventral midline (arrow), seen at higher magnification in J' (arrow). K) Stage 13, dorsal view showing salivary glands (s). L) Stage 5 blastoderm, showing apical capping of dSTIM protein, association of dSTIM with lateral cell membranes (M, N), and presence of dSTIM in pole cells (arrow) (O). P) Stage 15, showing dSTIM localization in clusters of CNS cells along the ventral midline (arrow) and salivary glands (s) (Q).

To detect *dSTIM *RNA expression during embryonic development we performed whole mount *in situ *hybridization using DIG-labelled RNA probes (Fig. 1A–K). *dSTIM *RNA was maternally deposited in the *Drosophila *egg (Fig. 1A), with transcript levels decreasing as development proceeded through stages of early nuclear division, pole cell formation, syncytial blastoderm and cellularisation (Fig. 1A–C). dSTIM transcripts appeared to be specifically excluded from pole cells (Fig. 1B, C). During gastrulation and towards the initiation of germ-band extension transcripts were concentrated within invaginating tissues, within the ventral and cephalic furrows (Fig. 1D, E). This was most likely the first evidence of zygotic expression. As the germ-band fully extended, *dSTIM *transcripts appeared to be expressed in the anterior and posterior midgut primordia, and dSTIM expression persisted in the midgut through germ-band retraction when the anterior and posterior midguts fuse (Fig. 1E–H). During these stages *dSTIM *RNA also appeared uniformly expressed at low levels in the ectoderm. At the start of dorsal closure *dSTIM *expression was maintained in the midgut and also appeared in the malphigian tubules, salivary glands and in specific cells clustered at intervals along the ventral midline within the central nervous system (CNS) (Fig. 1I–K). At later stages, approaching completion of embryonic development, *dSTIM *transcripts appeared to be uniformly expressed throughout the midgut, across the entire epidermis and in cell clusters in the CNS. At all stages, control hybridization with sense RNA probes produced negligible background staining (data not shown).

We then assessed the localization of dSTIM protein by immunofluorescent staining and observed similar expression patterns to those observed with *in situ *hybridization (Fig. 1). dSTIM first appeared to be associated with newly formed cell membranes of the blastoderm stage embryo, over the entire cell surface of each cell (Fig. 1L–O). The localization of dSTIM appeared more intense at the apical capping of cells, but uniform around the lateral and basal membranes. Cytoplasmic dSTIM was detected in pole cells (Fig. 1O), in contrast to the absence of *dSTIM *transcripts in these cells (Fig. 1C, D). At later stage embryos, dSTIM was most evident along the ventral midline in cells situated at regular intervals between the anterior and posterior commissures of the CNS (Fig. 1P), corresponding to the position of glial cells. High levels of dSTIM were also detected in cells of the salivary gland (Fig. 1Q). Embryos stained with peptide-blocked antibody were negative, demonstrating the specificity of the immunolocalization (data not shown).

### dSTIM expression in larval tissues

**Figure 2:**
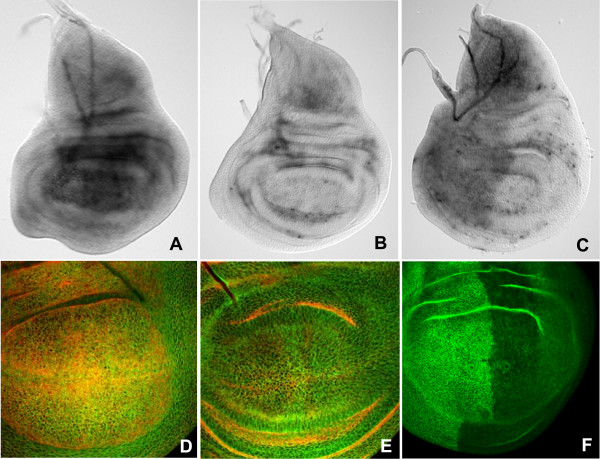
**RNAi effectively knocks down dSTIM in wing imaginal discs**. Whole mount *in situ *RNA hybridization (A-C) and immunofluorescence (D-F) to detect dSTIM expression in 3^rd ^instar wild-type wing (A, B, D, E) and wing discs expressing *dSTIM*^*RNAi *^induced by *en.Gal4 *(C, F). Discs are presented with anterior end to the left and dorsal end at the top. Hybridisation with the anti-sense RNA probe shows uniform expression of *dSTIM *transcripts in the wing disc (A). Hybridisation with the sense probe shows negligible staining throughout the wing discs, except for trapping of probe in tissue folds (B). dSTIM protein (green) is uniformly expressed both apically (D) and basally (E) in cells of the wing disc, in contrast to Arm (red) which is mainly apically localized (D, E). Expression of *dSTIM*^*RNAi *^driven by *en.Gal4 *results in a marked reduction in transcript (C) and protein (F) levels confined to the posterior region of the wing disc.

During the late stages of larval growth and development (3^rd ^instar), *dSTIM *transcripts were uniformly expressed in wing, eye and leg imaginal discs. In the wing disc *dSTIM *transcripts were evenly distributed, with no apparent preferential localization to specific compartments, boundaries or regions (Fig. 2A). While hybridization appeared more intense within tissue folds, similar staining with sense RNA probes demonstrated this to be a non-specific artifact (Fig. 2B). Similar uniform patterns of expression were observed in the eye and leg discs (data not shown). By immunostaining we found that dSTIM was uniformly expressed in the wing discs, where it was found both at the apical and basal surfaces of the columnar epithelial cells, with no evidence of a polarized distribution (Fig. 2D, E). This non-polarised distribution is in contrast to that of Armadillo (Arm) which is localized to apical adherens junctions (Fig. 2D, E) [40], or discs large (Dlg), which is primarily associated with the apico-lateral septate junctions (data not shown) [41]. Wing discs incubated with peptide-blocked antibody exhibited negligible background staining, confirming the specificity of immunolocalization of dSTIM (data not shown).

### Validation of dSTIM^RNAi ^and dSTIM^FL ^expression in transgenic flies

To study the role of dSTIM in the fly we generated and analyzed two independent *P{UAS-dSTIM*^*RNAi*^*} *transformant lines. To determine whether *dSTIM*^*RNAi *^could efficiently and specifically interfere with *dSTIM *transcripts, the level of dSTIM expression was analyzed by *in situ *hybridization and immunofluorescence in third instar wing imaginal discs expressing *dSTIM*^*RNAi *^driven by *en.Gal4 *[42,43]. Markedly reduced levels of *dSTIM *transcripts and protein were seen within the posterior wing compartment compared to the anterior compartment expressing endogenous levels of dSTIM, which corresponded to the known expression domain of *engrailed *(Fig. 2C, F). Substantial reduction of *dSTIM *trancripts was also observed in the stage 10 embryo expressing *dSTIM*^*RNAi *^driven by *en.Gal4*, with loss of expression in ectodermal stripes corresponding to sites of *engrailed *expression (data not shown). In addition, transient co-expression of *dSTIM*^*RNAi *^with *dSTIM*^*FL *^in S2 cells reduced expression of dSTIM protein to background levels as measured by Western blotting (data not shown). These data provide evidence that *dSTIM*^*RNAi *^was expressed in the correct domain in transgenic embryos and caused substantial posttranscriptional interference with *dSTIM*. Analysis of the effects of *dSTIM*^*RNAi *^expression in the wings of flies lacking one functional copy of dSTIM (see later) showed that the level of RNAi-mediated silencing was not equivalent to a complete knockout of gene expression but represented a substantial knockdown. No off-target 19-mer matches were detected when the 395 bp dsRNA sequence was analysed using the dsRNA verification algorithm at . Moreover, adult patterning defects produced from *en*.Gal4 and *sd*.Gal4 (data not shown) driven expression of *dSTIM*^*RNAi *^could be completely rescued by co-expression of *dSTIM*^*FL*^, indicating that observed phenotypes were the result of specific reduction in dSTIM levels.

For analysis of dSTIM overexpression, four independent *P{UAS-dSTIM*^*FL*^*} *transformant lines were generated and characterized. Analysis of *dSTIM*^*FL *^expression using the *en.Gal4 *driver showed dSTIM transcripts and protein were significantly upregulated in the posterior wing compartment (data not shown).

### Modulating dSTIM expression in early development

#### RNAi-mediated knockdown

To study the effect of *dSTIM*^*RNAi *^during embryogenesis we used the *da.Gal4 *and *e22c.Gal4 *drivers to induce ubiquitous expression of *RNAi *in the embryo. This resulted in late 2^nd ^to early 3^rd ^instar larval lethality, with considerable variability between the progeny. Larvae appeared contracted and not highly active, but detailed examination indicated they were normal in size with normal patterning of denticle belts. An obvious characteristic of all transgenic larvae was the transparency of fat body cells, suggestive of inadequate nutrition or storage of nutrients. The specific cause of larval immobility and death was not determined.

#### Transgenic overexpression

The *da.Gal4 *and *e22c.Gal4 *drivers were also used to analyze the effects of *dSTIM*^*FL *^overexpression during embryogenesis. Embryos expressing *dSTIM*^*FL *^did not survive beyond the early 3^rd ^instar larval stage, with lethality at a similar stage to embryos expressing *dSTIM*^*RNAi*^. These data indicate that neither overexpression nor reduction of dSTIM expression in the early embryo cause major problems with embryonic patterning but most likely cause more subtle defects in muscle or neural development necessary for nutrition that are incompatible with larval hatching and survival. We did not ascertain whether the same cell lineages were affected in embryos expressing *dSTIM*^*FL *^and in those expressing *dSTIM*^*RNAi*^.

### Modulating dSTIM expression in the eye

#### RNAi-mediated knockdown

Since dSTIM is highly expressed in the eye imaginal disc, we firstly used the *ey.Gal4 *driver to induce expression of *dSTIM*^*RNAi *^during early specification and growth of the eye disc [44]. Expression of *dSTIM*^*RNAi *^resulted in no observable phenotypic abnormalities in eye development (data not shown), indicating that the levels of endogenous dSTIM expression are not essential for the proper patterning and development of the eye, or that sufficient dSTIM is still translated to perform its normal function during eye development.

We next used three Gal4 drivers to induce expression of *dSTIM*^*RNAi *^in different cell populations during later stages of eye development: *GMR.Gal4 *to drive expression in all disc cells formed behind the morphogenetic furrow,* lz.Gal4 *to drive expression in cone and pigment cells and photoreceptors, and *sca.Gal4 *to drive expression in sensory organ precursors [45-47]. Expression of *dSTIM*^*RNAi *^using any of these drivers had no observable effects on eye phenotype (data not shown).

#### Transgenic overexpression

**Figure 3:**
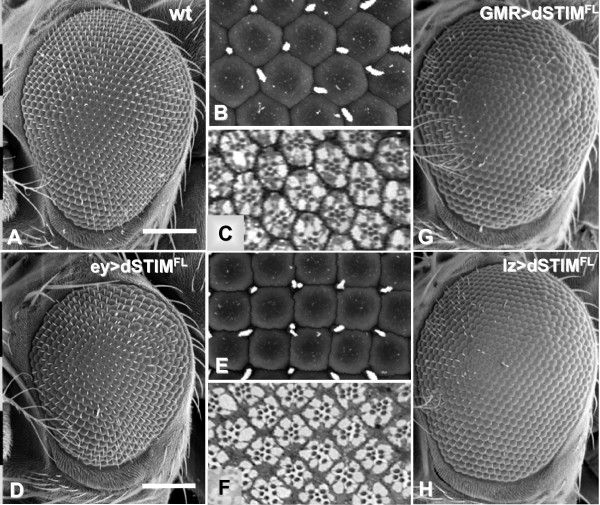
**Ectopic expression of dSTIM in eye imaginal discs results in small eyes with tetragonal packed ommatidia**. Scanning electron micrographs (SEM) of adult eyes in wild-type (wt) flies (A, B) and in flies expressing *dSTIM*^*FL *^(D, E) driven by *ey.Gal4*. Scale bars in A and D represent 100 μm. Eyes expressing *dSTIM*^*FL *^are smaller than wild-type eyes, and at higher magnification are seen to have a square-shaped ommatidial lattice (E) compared to the hexagonal lattice in wild-type eyes (B). Tangential sections reveal no underlying abnormalities in the specification of photoreceptor clusters (C, F). SEM of adult eyes overexpressing *dSTIM*^*FL *^driven by *GMR.Gal4 *(G) and *lz.Gal4 *(H) show a 'shaven' phenotype in both cases, characterized by loss of interommatidial bristles across most of the eye and mild roughening of the eye surface.

When *dSTIM*^*FL *^was overexpressed in the eye imaginal disc using the *ey.Gal4 *driver adult eyes were significantly smaller than those of wild-type flies (Fig. 3A, D), with abnormally shaped ommatidia (Fig 3B, E). Rather than the normal hexagonal lattice with bristles positioned at alternating vertices, the facets had a tetragonal (square) configuration with bristles at each corner. Some vertices had duplicated bristles due to the altered packing of the facets. Tangential sections through the eye revealed normal numbers and orientation of photoreceptors in each tetragonal ommatidial unit (Fig. 3C, F).

Overexpression of *dSTIM*^*FL *^at later stages using *GMR.Gal4*, *sca.Gal4 *and *lz.Gal4 *drivers had no visible effects on eye size, but resulted in loss of interommatidial bristles, giving rise to a 'shaven' phenotype (Fig. 3G, H). Interommatidial bristles were absent when *dSTIM*^*FL *^was expressed within the bristle progenitor cells under the influence of the *GMR.Gal4 *(Fig. 3G) and *sca.Gal4 *(data not shown) drivers, as well as when *dSTIM*^*FL *^was expressed in ommatidial cells but not in the bristle progenitors under the influence of the *lz.Gal4 *driver (Fig. 3H). These observations suggest that the effects of *dSTIM*^*FL *^overexpression are not restricted to a specific cell population within the developing eye. Flies expressing *dSTIM*^*FL *^driven by *GMR.Gal4 *and *lz.Gal4 *also had mild roughening of the eye (Fig. 3G, H), although tangential sections showed no abnormalities in the photoreceptor clusters that generally underlie a roughened phenotype (data not shown).

### Modulating dSTIM expression in the notum

**Figure 4:**
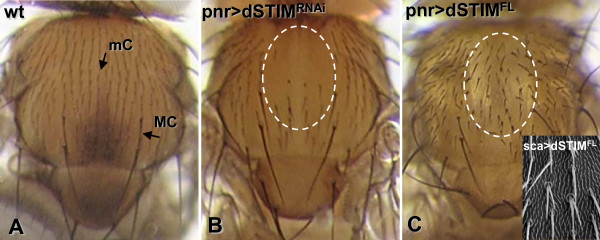
**dSTIM regulates mechanosensory bristle differentiation**. Dorsal view of the adult wild-type (wt) notum (A), showing the position and orientation of single michrochaete (mC) and macrochaete (MC) mechanosensory bristles. Flies expressing *dSTIM*^*RNAi *^driven by *pnr.Gal4 *exhibit a partially bald cuticle lacking mC over the *pannier *expression domain (dotted ring) (B), while flies overexpressing *dSTIM*^*FL *^exhibit misorientated and duplicated bristles when induced by *pnr.Gal4 *(C), or *sca..Gal4 *(inset C).

We analyzed the role of dSTIM in the development of the notum by inducing expression of *dSTIM*^*RNAi *^and *dSTIM*^*FL *^in the mesothoracic disc using the *pnr.Gal4 *driver. Expression of *dSTIM*^*RNAi *^resulted in a partially bald notum, with loss of mechanosensory bristles (Fig. 4B). Microchaetes were missing from the domain of highest *pannier *expression [48], while macrochaetes, which develop later than machrochaetes, appeared to be largely unaffected. In contrast, overexpression of *dSTIM*^*FL *^driven by *pnr.Gal4 *and *sca.Gal4 *in sensory organ precursors and *MS1096.Gal4 *(data not shown) produced misorientated and twin-shafted mechanosensory bristles (Fig. 4C), with microchaetes again being mostly affected. Thus *dSTIM*^*RNAi *^and overexpression of dSTIM have opposite effects on the development of mechanosensory bristles.

### Modulating dSTIM expression in the wing

To determine the role of dSTIM in patterning of the fly wing several Gal4 drivers were used to target expression of *dSTIM*^*RNAi *^and *dSTIM*^*FL *^in different compartments of the developing wing: *en.Gal4 *in the posterior wing compartment (see Fig 2); *MS1096.Gal4 *in the dorsal compartment of the wing pouch [49]; *sd.Gal4 *in the entire wing pouch [50], *32B.Gal4 *in the entire wing disc [39], and *sca.Gal4 *in sensory organ precursors.

#### RNAi-mediated knockdown

**Figure 5:**
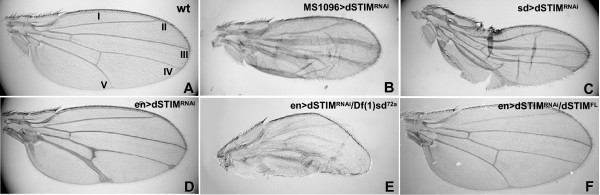
**dSTIM regulates wing vein differentiation**. Patterning of wing veins in adult wild-type (wt) wings (A), and wings expressing *dSTIM*^*RNAi *^induced by *MS1096.Gal4 *(B), *sd.Gal4 *(C) and *en.Gal4 *(D). The position of lateral veins I, II, III, IV and IV is shown in A. Thickened veins are present within the expression domains of each *Gal4 *driver, with no defects at the wing margins. Expression of *dSTIM*^*RNAi *^driven by *en.Gal4 *in a heterozygous *dSTIM *deficiency background (*Df(1)sd*^72*a*^) results in enhanced vein thickening (E) when compared to (D). Co-expression of *dSTIM*^*RNAi *^with *dSTIM*^*FL *^driven by *en.Gal4 *results in complete rescue of the thick vein phenotype (F).

We observed that expression of *dSTIM*^*RNAi *^induced by all Gal4 drivers except *sca.Gal4 *caused thickening of wing veins, with no other patterning abnormalities being evident (Fig 5A–D). The severity of the phenotype was increased by either the copy number of transgenes, or when the temperature was increased to 25°C or to 29°C to enhance Gal4 activity (data not shown). Expression of *dSTIM*^*RNAi *^in the dorsal wing pouch using the *M1096.Gal4 *driver produced the most severe phenotype, with pronounced thickening of veins III, IV and V (Fig. 5B). Vein thickening was confined to the posterior wing when *en.Gal4 *was used to drive *dSTIM*^*RNAi *^(Fig. 5D). Vein thickening was associated with an apparent reduction in wing blade size, most evident when expression of *dSTIM*^*RNAi *^was induced with *MS1096.Gal4 *and *sd.Gal4 *drivers (Fig. 5B, C). The smaller wing size may be the result of inadequate wing inflation [51].

To determine whether the effects of RNAi were hypomorphic or nullimorphic, we tested the effects of *dSTIM*^*RNAi *^induced with the *en.Gal4 *driver in a *dSTIM *hemizygous background using the *Df(1)sd*^72*a *^strain which expresses only one copy of dSTIM. We observed a severely enhanced 'thick vein' phenotype (Fig 5E compared to 5D), demonstrating that while *dSTIM*^*RNAi *^caused specific and efficient posttranscriptional silencing of *dSTIM *(Fig. 2) the level of silencing was not equivalent to a complete 'knockout', but rather represented a 'knockdown'. The 'thick vein' phenotype observed with *dSTIM*^*RNAi *^was completely reversed when *dSTIM*^*RNAi *^was co-expressed with *dSTIM*^*FL *^driven by *en.Gal4 *(Fig. 5F), further demonstrating that the observed silencing correlates with a "knockdown' effect, but also validating that re-expression of dSTIM rescues the phenotype observed consequent to reduced dSTIM levels.

#### Transgenic overexpression

**Figure 6:**
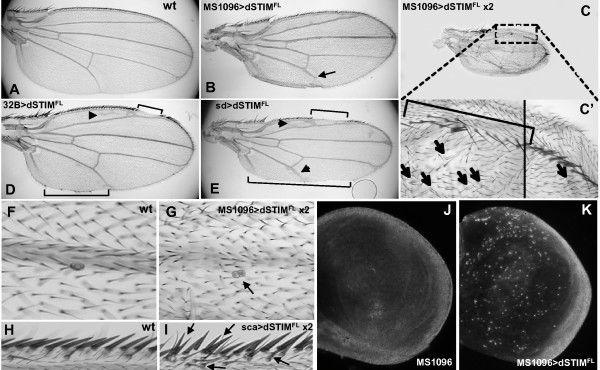
**Ectopic dSTIM results in smaller wings due to increased apoptosis and affects vein and bristle differentiation**. Patterning of the adult wild-type (wt) wing (A) and wings expressing *dSTIM*^*FL *^induced by various *Gal4 *drivers (B-K). Overexpression of *dSTIM*^*FL *^induced by *32B.Gal4 *(D) and *sd.Gal4 *(E) result in wing margin defects seen as notching at the wing margin (brackets) and formation of ectopic veins (arrowheads). Overexpression of *dSTIM*^*FL *^driven by *MS1096.Gal4 *results in smaller wing size, with no apparent margin defects, but loss of the distal end of vein V (arrow) (B). Expression of two transgene copies of *dSTIM*^*FL *^driven by *MS1096.Gal4 *results in a further reduction in wing blade size (C). Loss of some mechanosensory bristles along the anterior wing margin (bracket) and multiple wing hairs (arrows) are evident at higher magnification (C'). Duplication of occasional campaniform sensilla (arrow) (G) and duplication of margin bristles are evident (arrows) (I) when two transgene copies of *dSTIM*^*FL *^are driven by *sca.Gal4*, that are not seen in wild-type wings (F, H). Staining of 3^rd ^instar larval wing discs with acridine orange shows few positive cells in *MS1096.Gal4 *discs (J), and a high number of positively stained cells in wing discs expressing *dSTIM*^*FL *^induced by *MS1096.Gal4 *(K), indicating an increased rate of apoptosis.

We next analyzed the effects of overexpressing *dSTIM*^*FL *^in the developing wing and observed significant wing margin defects with the *en.Gal4, sd.Gal4 *and *32B.Gal4 *drivers (Fig. 6). When expression of *dSTIM*^*FL *^was induced by *en.Gal4 *wings displayed regions of margin loss (notching) confined to the posterior wing margin (data not shown), while both anterior and posterior wing margins were notched when expression was induced by *32B.Gal4 *and *sd.Gal4 *drivers (Fig. 6D, E). Whichever of these drivers was used, the margin at the distal wing tip was never compromised. Expression of *dSTIM*^*FL *^in the dorsal compartment driven by *MS1096.Gal4 *had no effect on the wing margin, but resulted in smaller sized wings (Fig. 6B), which were blistered as a result of uneven growth of the dorsal compartment relative to the ventral compartment which does not express *MS1096.Gal4*. Increasing the number of transgene copies or increasing the temperature to 29°C led to further reduction in wing size (Fig. 6C), loss of some mechanosensory bristles along the wing margin, and multiple wing hairs (mwh) (Fig. 6C') as well as duplication of campaniform sensilla (mechanosensory organs) (Fig. 6F, G). Duplication of margin bristles was observed when *dSTIM*^*FL *^expression was driven by two copies of *sca.Gal4 *(Fig. 6H, I). Some wings also exhibited irregular venation, with appearance of ectopic veins in the I-II region and deltas at the ends of veins IV and V (Fig. 6D, E). Distal loss of vein V was seen when expression was driven by *MS1096.Gal4 *(Fig. 6B). To determine whether reduced wing size of flies expressing *dSTIM*^*FL *^was due to increased cell death, 3^rd ^instar wing discs were stained with acridine orange. Limited cell death was seen in control *MS1096.Gal4 *wing discs, while cells in wing discs expressing *dSTIM*^*FL *^driven by *MS1096.Gal4 *showed extensive acridine orange staining (Fig. 6J, K), indicative of an increased rate of apoptosis.

### Genetic interactions between dSTIM and Notch signaling in the wing

**Figure 7:**
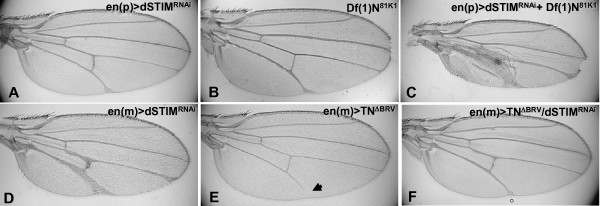
**Genetic interaction suggests that dSTIM positively supports Notch signalling during the refinement of vein thickness**. Wings expressing *dSTIM*^*RNAi *^in the posterior compartment induced by *en.Gal4 *(A, D). have thickened lateral veins IV and V. This phenotype is more severe in (D) than that of (A), since the *en.Gal4 *driver was introduced maternally (*en(m)*) in (D), while it was introduced paternally (*en(p)*) in (A). Wings from flies deficient in Notch expression (*Df(1)N*^81*k*1^) (B) have margin defects at the distal wing tip, moderate thickening of vein V, and deltas at the ends of veins (B). Expression of *dSTIM*^*RNAi *^induced by *en.Gal4 *on a *Notch *deficiency background results in severe exaggeration of vein IV and V thickening (C) when compared to *dSTIM*^*RNAi *^on a wild-type background (B), with no enhancement of margin defects. Note: in this instance (C), the *en.Gal4 *driver was introduced paternally, since *Df(1)N^81K1 ^*hemizygous males are lethal. Expression of an activated Notch allele (TN^ΔBRV^) induced by *en.Gal4 *(E) causes a reduction in the length of lateral vein V (arrowhead). Co-expression with *dSTIM^RNAi ^*(F), results in a mutual suppression of phenotypes caused by the expression of each construct alone. Note: The wings presented in (E) and (F) should be compared to (D), since the *en.Gal4 *driver was introduced maternally.

The Notch signaling pathway plays an important role in developmental processes of the fly notum, eye and wing that were influenced by altering dSTIM expression levels. In the wing, Notch signaling specifies both the patterning of vein thickness and the development of the wing margin. We thus investigated whether dSTIM interacts genetically with Notch in wing development. We determined whether Notch signaling is suppressed in the dSTIM transgenic wings, by analyzing the effects of *dSTIM*^*RNAi*^and *dSTIM*^*FL *^expression in *Df(1)N*^81*k*1 ^flies that lack one functional *Notch *allele. Flies hemizygous for *Notch *(*Df(1)N*^81*k*1^) displayed mildly thickened veins, deltas at the distal ends of wing veins and margin defects particularly at the distal tip (Fig 7B). This vein patterning phenotype was essentially identical to *dSTIM*^*RNAi *^driven by *en.Gal4 *introduced from the male parent (Fig 7A), and less severe than when introduced from the female parent (Fig 7D). RNAi-mediated knockdown of dSTIM driven by *en.Gal4 *on the *Df(1)N*^81*k*1 ^background significantly enhanced vein thickening in the posterior region of the wing but had no additional effects on patterning of the wing margins (Fig. 7C). Expression of a constitutively activated Notch allele (TN^ΔBRV^) induced by a maternally introduced *en.Gal4 *caused a reduction in the length of lateral vein V (Fig 7E). Co-expression of TN^ΔBRV ^with *dSTIM*^*RNAi *^resulted in mutual suppression of both phenotypes (Fig 7D–F). Overexpression of dSTIM^*FL *^on the *Df(1)N*^81*k *^background significantly enhanced margin loss (data not shown).

These data demonstrate that the effects of RNAi-mediated knockdown of dSTIM on wing vein thickness and transgenic overexpression of dSTIM on wing margin loss are both enhanced in a *Notch *deficiency background, indicating a potential genetic interaction between dSTIM and Notch in wing development

### Effects of dSTIM expression on Wingless signaling

The patterned adult wing originates along the dorso-ventral (D/V) boundary, which is specified through activation of Notch at the interface between the dorsal and ventral compartments of the wing disc [52,53]. Notch activates Wingless (Wg) and other genes along the D/V boundary [54-56] and Notch and Wg are required together to activate Cut, a regulator of sensory organ identity in the boundary cells at the wing margin [57]. Secreted Wg that is produced in the D/V cells diffuses across the neighbouring dorsal and ventral compartments forming a morphogenetic gradient that activates various downstream outputs, such as the proneural gene Achaete (Ac) in the sensory organ precursors (SOPs) bordering the D/V boundary, and distal-less (Dll), a regulator of wing growth [58]. Wg also stabilizes cellular levels of Armadillo (Arm), the major transcriptional regulator activated by canonical Wg signaling [59]. To determine whether dSTIM overexpression causes wing margin defects by influencing Wg signaling, we analyzed the effects of *dSTIM*^*FL *^expression on activation of these Wg signaling outputs.

**Figure 8:**
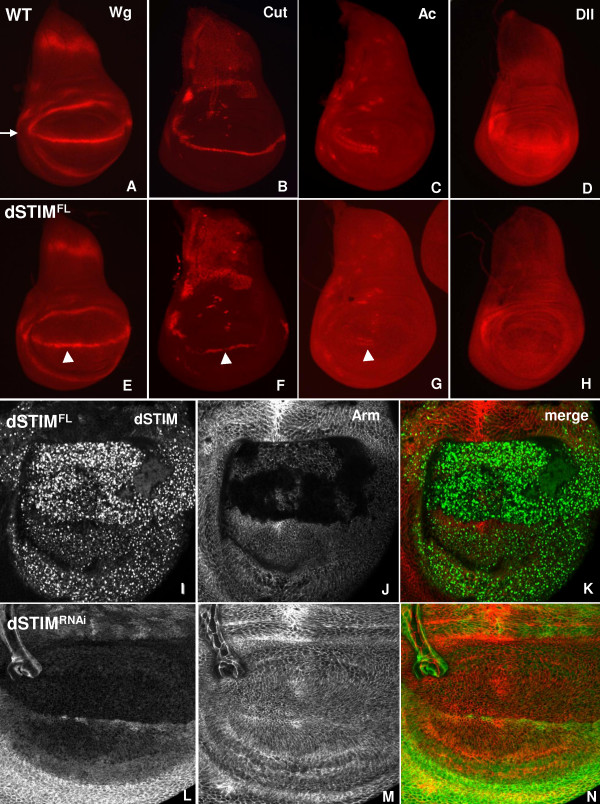
**dSTIM has downstream effects on Wg signalling**. Immunofluorescent staining of 3^rd ^instar larval wild-type (A-D) wing discs and discs expressing *dSTIM*^*FL *^induced by *sd.Gal4 *((E-H), with antibodies to detect Wg (A, E), Cut (B, F), Ac (C, G) and Dll (D, H). Expression of each protein is reduced at the dorsal/ventral boundary (arrow) except for expression of Wg, Cut and Ac in the central area close to the anterior-posterior boundary (arrowheads). Immunofluorescent staining of basal areas of wing discs expressing *dSTIM*^*FL *^(I-K) and *dSTIM*^*RNAi *^(L-K) induced by *MS1096.Gal4 *with antibodies to detect dSTIM (I, L) and Arm (J, M). Merged images show dSTIM (green) and Arm (red) (K, N). Expression of Arm is markedly reduced in cells overexpressing dSTIM^FL ^(J, K), but is not affected by expression of *dSTIM*^*RNAi *^(M, N).

Immunostaining was used to detect expression of Wg, Cut, Ac and Dll in 3^rd ^instar wing discs isolated from wild-type flies and flies expressing *dSTIM*^*FL *^driven by *sd.Gal4 *(Fig. 8). A marked reduction in the thickness of the band of D/V boundary cells expressing Wg was observed in wing discs expressing *dSTIM*^*FL *^(Fig. 8A, E), and Cut expression was also reduced at the outer margins of the discs (Fig. 8B, F). In addition, fewer Ac-expressing cells were seen, reflecting the reduced number of sensory bristles in the adult wings (Fig. 8C, G). Expression of Wg, Cut and Ac was not affected in the central region at the D/V margin (Fig. 8), an area that develops into the distal wing tip margin unaffected by *dSTIM*^*FL *^overexpression. A marked reduction in Dll expression was also evident in the dorsal and ventral compartments of wing discs expressing *dSTIM*^*FL *^(Fig. 8D, H). Overexpression of *dSTIM*^*FL *^driven by *MS1096.Gal4 *caused a significant reduction in the levels of cytoplasmic Arm (Fig. 8I–K). These results show that all Wg signaling outputs are reduced at the D/V margin when dSTIM is overexpressed. We could not detect any effect on Arm expression in wing discs expressing *dSTIM*^*RNAi*^, which is consistent with the absence of margin defects in the adult wing (Fig. 8L–N).

## Discussion

These studies demonstrate that while dSTIM is ubiquitously expressed in *Drosophila *embryonic and larval tissues, these endogenous levels of dSTIM expression are not essential for many developmental processes. RNAi-mediated posttranscriptional knockdown of dSTIM expression in the early embryo had no observable effects on growth or patterning of larvae, but did cause lethality by early 3^rd ^instar stages. Reduced mobility of larvae suggested functional defects in myogenic and/or neurogenic lineages that could affect nutrient intake and metabolism. This late embryo lethal phenotype was also observed when dSTIM was overexpressed at early stages, indicating that normal tissue function is dependent on the maintenance of correct levels of dSTIM expression and thus highly regulated Ca^2+ ^entry at these stages. STIM1-deficiency in mouse embryos influences the development and contractile function of skeletal muscle [33], and deficiencies in either STIM1 or STIM2 cause perinatal lethality but do not interfere with early processes of embryonic growth and development [32]. Likewise, STIM1-knockdown in the *C. elegans *nematode does not result in embryonic lethality, but reduces the contraction of sheath cells that trigger spermathecal opening for ovulation, and results in complete sterility [60]. Together these studies indicate that many cell types in developing invertebrate and mammalian embryos are not reliant on high levels of STIM expression for survival, proliferation and differentiation, and may regulate Ca^2+ ^influx through STIM-independent mechanisms. Since RNAi did not cause complete knockout of dSTIM gene expression generation of flies having a null mutation in *dSTIM *will be necessary to determine the absolute requirement of dSTIM in developing *Drosophila *tissues, as will an analysis of the phenotype of double STIM1/STIM2 knockout mice.

Using RNAi-mediated knockdown in imaginal discs we have shown that dSTIM does have an essential and very specific developmental role in tissue patterning and cell fate specification in the wing and the notum, but not in the eye. Absence of an eye phenotype could indicate either inadequate knockdown of dSTIM expression with any of the *Gal4 *drivers used or that dSTIM is indeed not required for normal eye development. Knockdown of dSTIM expression in the developing wing resulted in significant vein thickening with no other observable patterning defects, while dSTIM-deficiency in the mesothoracic disc resulted in loss of mechanosensory bristles (microchaetes) on the notum. Notch signaling plays a key role in these two independent developmental processes and we have demonstrated a genetic interaction between dSTIM and Notch function in the wing. Notch signaling normally acts to prevents cells adopting a vein cell fate by inhibiting activation of the Egfr pathway and subsequent activation of the Dpp pathway [61], such that inhibition of Notch signaling promotes the differentiation of provein territory into vein tissues and gives rise to abnormally thickened veins [62,63]. We observed enhancement of vein thickening when Notch deficiency was combined with dSTIM knockdown, and showed that the activated Notch allele, *TN*^*ΔBRV*^, an allele known to rescue most Notch hypomorphic alleles [64], was able to suppress the RNAi-mediated dSTIM deficiency. These results suggest that dSTIM function specifically intersects with regulators of Notch signaling during regulation of vein differentiation.

The phenotypic effects of dSTIM knockdown and dSTIM overexpression in the mesothorax indicate a role for dSTIM in cell fate specification in the mechanosensory bristle cell lineage, a process also regulated by Notch signaling. The absence of mechanosensory bristles (microchaetes) on the notum in flies expressing *dSTIM*^*RNAi *^is consistent with a neurogenic phenotype, representing preferential neuronal specification of both daughter cells from the sensory organ precursor (SOP) [65]. Overexpression of dSTIM resulted in exactly the opposite phenotype, with the development of twin-shafted or duplicated bristles. While the underlying cellular basis for these phenotypes was not ascertained in this study, the data are consistent with defects either in the asymmetric division of SOP cells, or the process of lateral inhibition that specifies SOP cells within each proneural cluster in the notum, both of which are principally regulated by Notch signaling [65]. Loss-of-function mutations in the Notch receptor and in positive regulators of Notch cause a 'bald' neurogenic phenotype due to aberrant division and specification of daughter cells [66,67]. A similar neurogenic phenotype is caused by gain-of-function mutations in the Notch antagonist, *Numb*, while loss-of-function mutations in *Numb *result in the formation of duplicated bristles [68]. Asymmetric segregation of Numb during SOP cell division determines the level of Notch signaling in the daughter cells [69]. Numb is a target of the Ca^2+ ^-dependent kinase CamKII [70], and its polarized distribution has been shown to be regulated by aPKC phosphorylation [71], suggesting that Numb localization and function may be regulated by Ca^2+ ^-dependent mechanisms. Our data suggest that STIM-mediated Ca^2+ ^entry may potentially influence the cellular localization and/or activity of Numb or other Notch regulators. It is interesting that Notch signaling in mammalian cells has been linked to a calcineurin-dependent mechanism that activates NFAT [72], a pathway that is regulated by mammalian STIM1 [32,33]. While Ca^2+ ^-regulated NFAT homologues have not been identified in *Drosophila *[73], genetic screens have identified dSTIM as a regulator of nuclear translocation and dephosphorylation of NFAT [12] and *Drosophila *calcineurin homologues regulate other target proteins, such as Egfr [74]. Our results suggest that Notch and dSTIM may intersect at a functional level through activation of calcineurin independently of NFATc proteins in *Drosophila*, and further studies are now required to elucidate the molecular nature of these interactions.

Apart from development of the mechanosensory bristle cell lineage in the mesothorax, overexpression of dSTIM did not have opposite developmental effects to those of dSTIM deficiency. DSTIM overexpression caused the misorientation of the ommatidial lattice in the eye and inhibited the formation of interommatidial bristles, and disrupted development of the wing margin, processes that were not influenced by dSTIM deficiency but are known to be regulated by Notch signaling. A secreted dominant-negative form of the Notch ligand Serrate (SerS), and the glycosyl transferase Fringe (Fng) are both negative regulators of Notch signaling, and their expression in the early eye results in reduced eye size and square rather than hexagonal ommatidial facets [75,76]. In addition, overexpression of SerS in the wing results in wing margin loss and downregulation of Wg and Cut at the wing margin [76]. Loss-of-function mutations in either the Notch receptor or in activators of Notch signaling result in loss of interommatidial bristles in the eye [77]. The similarity of these eye and wing phenotypes to those resulting from dSTIM overexpression support the proposal that dSTIM overexpression negatively regulates Notch signaling at these sites.

The developmental defects associated with dSTIM overexpression are also consistent with dominant effects of abnormally increased intracellular Ca^2+ ^levels causing enhanced activity of Ca^2+^-dependent processes. A major effect of increased intracellular Ca^2+ ^in many cell types is the induction of apoptotic cell death [5,78], and smaller eyes and wings were seen in flies overexpressing dSTIM, with increased apoptotic cell death observed in the wing disc. Observations of increased growth arrest and apoptosis in some mammalian cell lines overexpressing STIM1 led to the initial hypothesis that STIM1 functions as a tumour suppressor [31,79]. In addition to induction of apoptosis we have demonstrated that wing margin defects caused by overexpression of dSTIM in the wing disc were associated with decreased expression of Arm (β-catenin) and other Wg signalling outputs at the wing margin, demonstrating inhibition of canonical Wg signaling. While these effects may simply reflect a dependence on Notch activity at the wing margin [54-56], a direct interaction between dSTIM and Wg signaling is also possible. Intracellular Ca^2+ ^is an important second messenger in non-canonical Wg/Wnt signaling pathways that primarily modulate cell polarity and movement, including the vertebrate Wnt/Ca^2+ ^pathway that activates PKC and CamKII, and the planar cell polarity (PCP) pathway that activates Jun-N-terminal kinase (JNK) [80,81]. Induction of Ca^2+ ^release by non-canonical Wnt ligands activates PKC and CamKII enzymes that can antagonize components of β-catenin signaling [80], as observed in our study. The *Drosophila *PCP pathway plays a fundamental role in the orientation of hairs, bristles and ommatidia [82], and development of multiple wing hairs [83] which are all observed with overexpression of dSTIM. Planar polarity defects are generated by abnormal segregation or random dispersion of proteins, including the non-canonical Wg components Fz, and Dsh, that normally provide the cell with polarity cues [84]. These data support a potential role of dSTIM in providing Ca^2+ ^dependent mechanisms for determining asymmetric protein localization within the cell. While STIM proteins are major regulators of Ca^2+ ^entry through activation of SOC channels, there is increasing evidence for their role in store-independent Ca^2+ ^entry mechanisms [28,29]. Identification of the single *Drosophila *homologue of the SOC channel subunit protein Orai [12,38] will now allow a detailed analysis of SOC function during *Drosophila *development. Comparison of phenotypes caused by mutations in dSTIM and in dOrai will indicate whether the effects of dSTIM knockdown observed in this study are due specifically to a reduction in SOC-mediated Ca^2+ ^entry or whether dSTIM plays a role in developmental processes by mediating other pathways of Ca^2+ ^entry. Preliminary evidence from a genetic screen to identify molecules that affect vein patterning in the *Drosophila *wing suggests that ectopic expression of dOrai may have similar patterning effects (loss of vein and wing margin) to ectopic dSTIM [85].

Aberrant expression of STIM1 and STIM2 proteins has been associated with human cancer [79,86] but their direct role in tumorigenic events has yet to be established. However, a recent study has identified STIM2 as a putative target of β-catenin/TCF in colorectal cancer (A. Villanueva, personal communication). Deregulation of Ca^2+ ^homeostasis and Ca^2+^-mediated signaling events have been implicated in tumorigenesis [87], and using *Drosophila *as a model organism to determine how regulators of Ca^2+ ^homeostasis interact with developmental pathways such as Wnt and Notch will reveal potential mechanisms that can be further analysed in mammalian systems.

## Conclusion

The results presented here are the first to describe a developmental function of Ca^2+ ^entry mediated by dSTIM. While the precise molecular mechanisms cannot be elucidated from these studies we have identified Notch signaling as a potential pathway to be dependent on dSTIM-mediated Ca^2+ ^entry in certain cell types and Wg signaling to be influenced by dSTIM overexpression. The recent association of STIM2 with colorectal cancer also highlights the relevance of this family of proteins to molecular pathways that regulate cell proliferation and differentiation. A crucial aspect of Ca^2+ ^signaling is the necessity to generate localized transient elevations in Ca^2+ ^concentrations at important target sites within the cells. It is noteworthy that the developmental processes affected by alterations in dSTIM expression involve asymmetric cellular distribution of signaling components that establish spatial patterns and determine cell fate. The localization and translocation of STIM proteins in specialized regions of the ER place it in a good position to regulate highly localized Ca^2+ ^entry through the plasma membrane. Analysis of the co-localization of dSTIM activity with Ca^2+ ^dependent signaling will be a major step forward in determining whether dSTIM has an important role in regulating localized Ca^2+^-dependent signaling events that are critical for normal development, and can be aberrant in tumorigenesis.

## Methods

### Expression constructs and *Drosophila *transformation

To generate the *UAS-dSTIM*^*FL *^construct, encoding wild-type dSTIM, full-length *dSTIM *cDNA, from EST clone LD06112 (GenBank Acc. AA247009), was cloned into the pUAST vector [39] as a *Not*I-*Xho*I fragment. To generate the *UAS-dSTIM*^*RNAi *^construct used for inducing *in vivo *dsRNA interference, a genomic fragment and a cDNA fragment corresponding to the same region were amplified by PCR with primers containing unique restriction sites, and were sequentially cloned into the pUAST vector as an inverted repeat. The cDNA fragment was first amplified from LD06112, with the following primer set: 5'-CCGCTCGAGTTCCATGGGAAGTGGTTCAG-3' (Forward) and 5'-ATAAGAATGCGGCCGCTTGGCAAGGCAGCGCCAG-3' (Reverse), containing a *Xho*I and a *Not*I restriction site, respectively. The resultant PCR product was cut and cloned into the *Not*I/*Xho*I sites of pUAST to produce the intermediate vector pUAST/*dSTIM*^*ANDc*^. The genomic fragment was then amplified from *w*^1118 ^genomic DNA with the following primer set: 5'-GGAAGATCTGTTCCATGGGAAGTGGTTCAG-3' (Forward) and 5'-ATAAGAATGCGGCCGCCAATCTAAAACGCCGAAAATAG-3' (Reverse), containing a *Bgl*II and a *Not*I restriction site, respectively. It was cloned into the corresponding sites of pUAST/*dSTIM*^*ANDc *^resulting in the final *UAS-dSTIM*^*RNAi *^construct. To produce transgenic flies, each construct was injected into *w*^1118 ^embryos as described in [88].

### Fly stocks and genetics

Standard *Drosophila *stock maintenance and genetics procedures were followed throughout. All *Drosophila *lines, aside from those generated in this study (i.e. *UAS-dSTIM*^*FL *^and *UAS-dSTIM*^*RNAi*^), were obtained from either the sources indicated or from the Bloomington stock centre. The *w*^1118 ^strain was used as a wild-type control. The following Gal4 lines were used to drive expression of UAS lines: *scalloped.Gal4 *(*sd.Gal4*; [89], *MS1096.Gal4 *[49], *engrailed.Gal4 *(*en.Gal4*) [39], *32B.Gal4 *[39], *eyeless.Gal4 *(*ey.Gal4*) [90], *pannier.Gal4 *(*pnr.Gal4*) [48], *scabrous.Gal4 (sca.Gal4*) [47], *daughterless.Gal4 (da.Gal4) *[91] and *e22c.Gal4 *(Brand, 1997, personal communication to Flybase). The *Df(1)sd*^72*a*^/*Dp(1;Y)shi*^+^,*y*^+ ^strain containing a large deficiency of 13F1-4;14A [92] which includes *dSTIM*, and was used to generate flies hemizygous for *dSTIM*. The *Df(1)N*^81*k*1^,*v, [FRT101w*^+^]/*FM7c *strain containing a small deficiency of 3C5-6;3C9-10 including *Notch *[93] was used to generate flies hemizygous for *Notch*.

### *In situ *hybridization, immunocytochemistry and histology

*In situ *hybridization was performed on imaginal discs using DIG-labelled anti-sense and sense RNA probes as described previously in [94]. The RNA probes were synthesised from PCR products made using the following primer pairs that included a T7 promoter (underlined): 5'-TGGAGCAGGAAAATGTGGCAAC-3' (Forward) and 5'-GGATCCTAATACGACTCACTATAGGGCAGACCATTGTTGTTCACA-3' (Reverse) for the anti-sense, and 5'-GGATCCTAATACGACTCACTATAGGGAAGTGGTTCAGCGGATGGAGCGTG-3' (Forward) and 5'-GCCAAGTACATTGCCAACATAC-3' (Reverse) for the sense probe.

Immunostaining was performed according to standard protocols, with the following primary antibodies, used at the indicated dilutions: sheep affinity-purified anti-dSTIM (1:200 of 0.3 mg/ml) was custom made by Chiron Technologies (Clayton, Victoria), raised against the peptide, H-HRQLDDDDNGNIDLSESDDFLRC-NH_2_, dSTIM residues H151 to R172, corresponding to the EF-hand domain; mouse anti-Wg (1:500) [95]; rabbit anti-Dll (1:300) [96]; mouse anti-Ac (1:50) [97]; mouse monoclonal anti-Cut (1:200) [98]; and mouse anti-Arm (1:200) [99]. The anti-Wg, anti-Ac and anti-Arm antibodies were obtained from the Developmental Studies Hybridoma Bank (University of Iowa, USA). The anti-Dll and anti-Cut antibodies were provided by P. Whitington (University of Melbourne). Donkey anti-sheep IgG Alexa 488, donkey anti-mouse IgG 594 and donkey anti-rabbit Alexa 594 secondary antibodies, used at 1:500, were purchased from Molecular Probes. In peptide blocking experiments the anti-dSTIM antibody was incubated with a 100 molar excess of the immunogenic peptide (see above) for 1 hour prior to being applied to the tissue. Image acquisition was done either on a BioRad MRC1024 confocal microscope, or on a Zeiss Axioskop 2 fluorescence microscope, equipped with a Zeiss AxioCam HRc digital camera.

Adult wings were dissected and mounted on glass slides in a 1:1 ethanol/lactic acid solution. For colour imaging of the notum, whole adults were completely immersed in paraffin oil (to eliminate glare) and positioned on a bed of Sephadex-100 beads. Images were captured with a Dage-MTI DC330 3CCD camera mounted on a trinocular Zeiss Stemi SV/6 dissecting scope. Adult eyes were fixed in glutaraldehyde, embedded in Epon Araldite and sectioned as per standard histological protocols. Scanning electron micrographs of adult heads and notums were obtained as per standard protocols using a Philips SEM 515 electron microscope.

## Authors' contributions

JPE carried out and designed studies and participated in drafting the manuscript. AMA participated in design and analysis of studies. HR assisted in analysis of wing studies and drafting the manuscript. GRH and MD conceived the project, participated in its design and coordination, and drafted the manuscript.
